# Meta-analyses of comparative efficacy of antidepressant medications on peripheral BDNF concentration in patients with depression

**DOI:** 10.1371/journal.pone.0172270

**Published:** 2017-02-27

**Authors:** Chanjuan Zhou, Jiaju Zhong, Bin Zou, Liang Fang, Jianjun Chen, Xiao Deng, Lin Zhang, Xiang Zhao, Zehui Qu, Yang Lei, Ting Lei

**Affiliations:** 1 Department of Neurology, Yongchuan Hospital, Chongqing Medical University, Chongqing, China; 2 College of Computer and Information Science, Southwest University, Chongqing, China; 3 Institute of Life Sciences, Chongqing Medical University, Chongqing, China; 4 Children’s Hospital of Chongqing Medical University, Chongqing Medical University, Chongqing, China; 5 Department of neurology, University-town hospital of Chongqing Medical University, Chongqing, China; 6 Department of Physics, University of Fribourg, Fribourg, Switzerland; Chiba Daigaku, JAPAN

## Abstract

**Background:**

Brain derived neurotrophic factor (BDNF) is one of the most important regulatory proteins in the pathophysiology of major depressive disorder (MDD). Increasing numbers of studies have reported the relationship between serum/plasma BDNF and antidepressants (ADs). However, the potential effects of several classes of antidepressants on BDNF concentrations are not well known. Hence, our meta-analyses aims to review the effects of differential antidepressant drugs on peripheral BDNF levels in MDD and make some recommendations for future research.

**Methods:**

Electronic databases including PubMed, EMBASE, the Cochrane Library, Web of Science, and PsycINFO were searched from 1980 to June 2016. The change in BDNF levels were compared between baseline and post-antidepressants treatment by use of the standardized mean difference (SMD) with 95% confidence intervals (CIs). All statistical tests were two-sided.

**Results:**

We identified 20 eligible trials of antidepressants treatments for BDNF in MDD. The overall effect size for all drug classes showed that BDNF levels were elevated following a course of antidepressants use. For between-study heterogeneity by stratification analyses, we detect that length of treatment and blood samples are significant effect modifiers for BDNF levels during antidepressants treatment. While both SSRIs and SNRIs could increase the BDNF levels after a period of antidepressant medication treatment, sertraline was superior to other three drugs (venlafaxine, paroxetine or escitalopram) in the early increase of BDNF concentrations with SMD 0.53(95% CI = 0.13–0.93; *P* = 0.009).

**Conclusions:**

There is some evidence that treatment of antidepressants appears to be effective in the increase of peripheral BDNF levels. More robust evidence indicates that different types of antidepressants appear to induce differential effects on the BDNF levels. Since sertraline makes a particular effect on BDNF concentration within a short amount of time, there is potential value in exploring its relationship with BDNF and its pharmacological mechanism concerning peripheral blood BDNF. Further confirmatory trials are required for both observations.

## Introduction

Major depressive disorder (MDD) is a chronic mental illness that is characterized by persistent sadness and loss of interests for a minimum of two weeks [1–2]. Brain derived neurotrophic factor (BDNF) is a neurotrophic protein that has an important role in axonal growth, synaptic plasticity, and neuronal repair, and its levels are affected by stress [3–5]. There is growing evidence indicating that BDNF is related to many psychiatric disorders such as MDD in human studies [6–8].

Ever since Karege et al [9] demonstrated lower serum BDNF levels in depressed patients, considerable work has been carried out to investigate the relationship between decreased peripheral BDNF levels and pathogenesis of MDD [10–13]. Alternatively, several clinical studies pointed out that the down-regulation of peripheral BDNF levels in MDD patients could be reversed after a period of antidepressants (ADs) treatment. In parallel, earlier meta-analyses showed that peripheral BDNF levels were increased after pharmacological treatments [13, 14]. Moreover, several studies have investigated that ECT and different classes of antidepressants such as the serotonin selective reuptake inhibitors (SSRIs), serotonin norepinephrine reuptake inhibitors (SNRIs), and tricyclic antidepressants (TCAs) have effects on the peripheral BDNF in MDD patients [15–18]. However, whether all the antidepressants have effects on the BDNF levels remains controversial. For instance, Matrisciano et al found that both sertraline (an SSRI) and venlafaxine (an SNRI) could increase BDNF levels, while escitalopram (an SSRI) did not affect BDNF levels at either time point [19]. Başterzi et al. found that fluoxetine (an SSRI) changed the BDNF levels of the patient group with treatment, but venlafaxin could not [20]. Molendijk et al. previously observed that the use of SSRIs was related to the increase in serum BDNF level [10]. These results suggested that differential antidepressants may have variable effects on peripheral BDNF levels during treatment, which is still not well known.

Accordingly, BDNF may have potential use as a predictor of antidepressant efficacy, and abundant clinical data from pre-antidepressant and antidepressant-treated depressed patients motivated us to investigate the differential effects of antidepressant drugs on BDNF concentrations. We therefore performed a systematic review and meta-analysis aiming to examine the action of various reported antidepressant drugs on peripheral BDNF levels in MDD patients.

## Methods

The study design was performed according to the Preferred Reporting Items for Systematic Reviews and Meta-Analyses (PRISMA) Statement guidelines (S1 Text).

### Search strategy

Electronic databases (PubMed, EMBASE, the Cochrane Library, Web of Science, and PsycINFO) were systematically searched (S2 Text, which summarizes the applied search strategies). All previous human studies through June 2016 (published original clinical studies, conference proceedings, meta-analyses) comparing pre- and post-antidepressant treatment peripheral BDNF levels were considered. The search terms were settled as: (“brain derived neurotrophic factor” OR BDNF), “antidepressant”, AND (“major depression” OR “major depressive disorder” OR MDD OR “depressive episode” OR “depression”).

### Study selection strategy

Three independent researchers (TL, JJZ, and XD) selected studies for inclusion with discrepancies resolved by discussion. The titles and abstracts were scanned, and potentially relevant studies were reviewed in full. We included eligible studies examining plasma or serum BDNF levels in patients pre- and post-antidepressant treatment. Furthermore, each included study should make descriptions of the antidepressant use and time points in the therapy. Since we focused on the effect of pharmacological treatments on BDNF levels, studies using antidepressant treatment such as electroconvulsive therapy (ECT) or deep brain stimulation (DBS) were not suitable for this meta-analysis. Abstracts, case studies, family-based designs, population-based studies on healthy subjects, reviews, and duplicate cohorts were excluded.

### Data extraction and outcome measures

Three authors (TL, CJZ, and BZ) independently extracted data to avoid extraction errors. The following parameters were extracted from each eligible article: first author, publication year, country of origin, diagnostic system, number of subjects (male/female), antidepressants use, measuring sample (plasma or serum), BDNF levels, and Hamilton Depression Rating Scale (HDRS) scores. The primary outcome was BDNF levels from baseline to post-treatment and we extracted the data for short-term (1 to 12 weeks) in each study. If one study reported BDNF concentrations for more than one time point within our pre-defined periods, we considered the data recorded at baseline and the last time point within the range for the overall effect analysis.

### Statistical methods

First, all statistical analyses for meta-analysis were conducted using Rev Man 5.0.1 and STATA software (version 12.1; Stata Corporation, College Station, Texas, USA). All *P*-values were two-sided with a *P*<0.05 considered statistically significant. The alterations before and after antidepressant medication were assessed by SMDs (and their 95% CIs) for each study. A chi-squared-based Q-statistic test was used to detect the heterogeneity among studies, and a random-effects model was applied. We used a Z-test to determine the significance of the pooled SMDs with a *P*<0.05 considered statistically significant. Stratification-based analyses were carried out to evaluate heterogeneity and the possible moderating effects of between-study differences on outcomes.

In order to evaluate possible biases, a sensitivity analysis was conducted by leave–one-out methods to assess the contribution of each individual dataset to the pooled OR. Finally, we estimated publication bias by Egger’s test with a P<0.05 considered statistically significant.

## Result

**Fig 1:**
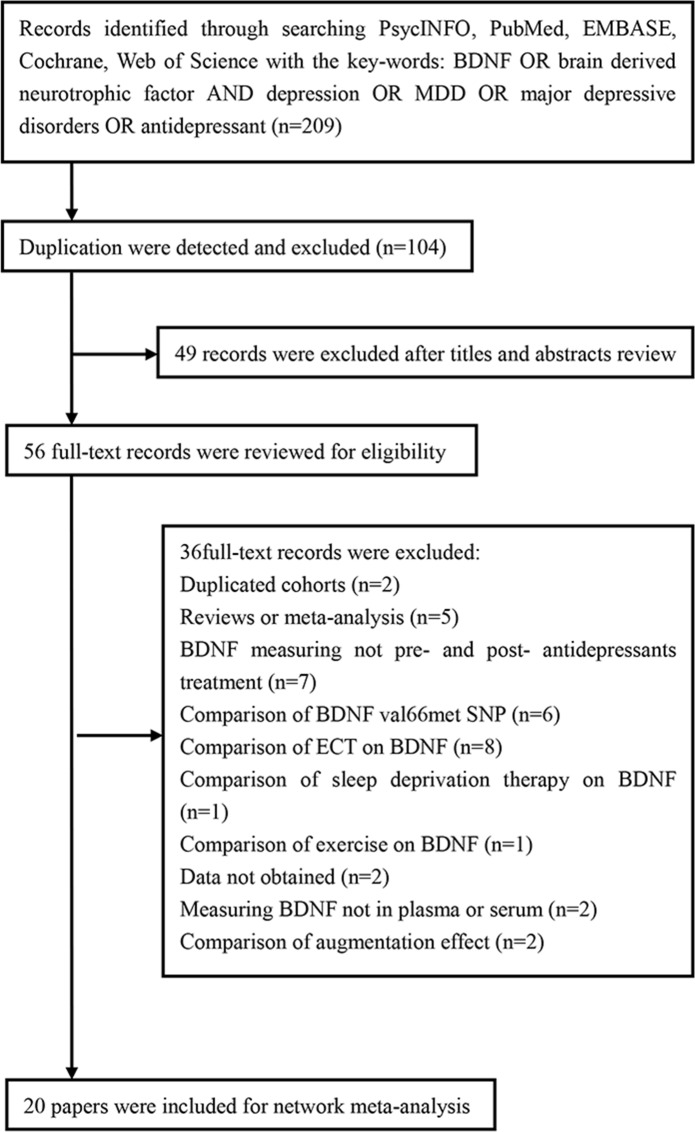
Flowchart of Study Selection.

The study selection procedure is shown in Fig 1 **(Fig 1)**. The literature search identified 206 potentially relevant records. After screening titles and abstracts, 56 full-text articles were reviewed, of which 36 were excluded for the following reasons: (i) two studies were duplicated cohorts; (ii) five studies were reviews or meta-analyses; (iii) seven studies did not assess BDNF pre- and post- antidepressant treatment; (iv) six studies compared the BDNF val66met SNP; (v) twelve studies did not compare antidepressant medication but analyzed effects of other methods(ECT, sleep deprivation therapy, exercise et al) on BDNF; (vi) data from two studies could not be obtained; (vii) two studies did not measure BDNF, either in plasma or serum. Hence, twenty studies were ultimately included in this meta-analysis based on our inclusion criteria [19, 21–39]. The study characteristics are displayed in Table 1. The characteristics of antidepressants such as antidepressants types, dose of antidepressants and duration of antidepressants treatment et al. are displayed in Table 2.

**Table 1:** Characteristics of 20 included studies (studies are sorted by year and month of publication).

Study	Year	Country	Diagnostic	Antidepressants	Number(F/M)	Mean age	BDNF			HDRS	
							Sample	Pre-treatment	Post-treatment	Pre-treatment	Post-treatment
Mikoteit	2015	Switzerland	DSM-IV	SNRI	25(8/17)	43.7±12.5	serum	5.01±3.64	7.3±5.6	22.2±4.9	9.0±6.6
Ghosh	2015	India	DSM-IV	SSRI,SNRI	60(32/28)	33.6±8.9	plasma	767.91±29.455	848.05±28.87	18.5±3.92	9.62±3.865
Yoshimura	2014	Japan	DSM-IV	SSRI	60	50.4±15.1	serum	8.6±7.2	11.2±8.4	21.0±4.3	10.0±5.6
Brunoni	2014	Brazil	MINI	SSRI	18	41.0±1.0	plasma	1331±786	1620±672	22.0±4.0	14.0±8.0
Martocchia	2014	Switzerland	DSM-IV	SSRI	5	74.0±6.8	serum	11.5 ± 0.6	16.0 ± 2.7	—	—
Munno	2013	Italy	DSM-IV	SSRI	16	48.19±10.3	serum	14.87±2.9	14.69±1.6	19.20±1.83	12.44±2.40
							plasma	231.4±90.1	312.9±109.4	19.20±1.83	12.44±2.40
Ladea	2013	Romania	DSM-IV	SSRI	20	36.6±8.1	serum	35.6±12.7	35.7±13.2	—	—
Deuschle	2013	Germany	DSM-IV	SNRI, Mirtazapine	56(41/15)	52.3±15.9	serum	7.73±6.99	7.84±5.49	22.85±4.35	8.15±5.65
Dreimüller	2012	Germany	DSM-IV	SSNRI, SNRI, TCA, Mirtazapine	39(19/20)	44.9±13.9	plasma	298±196	334±157	19	12
Wolkowitz	2011	USA	DSM-IV	SSRI	30(9/21)	39.1±9.6	serum	14.85±5.34	19.12±7.05	25.2±5.95	12.1±8.05
Rojas	2011	Chile	DSM-IV	SNRI	34(24/10)	41.05±11.15	serum	12.2±9.95	8.85±2.44	22.8±3.65	11.43±1.48
Matrisciano	2009	Italy	DSM-IV	SSRI,SNRI	21(10/11)	42.5±8.3	serum	35.37±14.33	39.43±14.97	17.6±5.3	—
Piccinni	2008	Italy	DSM-IV	SSNRI,SNRI,TCA	15	47.0±10.8	serum	19.3±8.8	24.457±9.921	—	—
							plasma	290±190	456.3±198.5	—	—
Huang	2008	China	DSM-IV	Mirtazapine. SSRI, SNRI	79(61/18)	37.3±10.0	serum	10.7±7.3	12.0±8.9	35.1±4.9	12.1±10.8
Hellweg	2007	Germany	DSM-IV	TCA,SSRI	20(17/3)	50.5±14.4	serum	13.13±4.43	13.60±5.41	23.4±4.5	11.35±8.25
Yoshimura	2007	Japan	DSM-IV	SSRI,SNRI	42(26/16)	46±20.5	serum	9.6±8.8	16.85±7.85	23.5±6.5	—
Aydemir	2006	Turkey	DSM-IV	SSRI	20(20/0)	35.55±7.58	serum	27.68±13.74	38.57±15.30	39.75±7.40	9.31±6.05
Gonul	2005	Turkey	DSM-IV	SSRI,SNRI	28(21/7)	35.5±8.1	serum	20.8 ± 6.7	33.3±9.89	27.28 ± 3.53	8.85±3.15
Aydemir	2005	Turkey	DSM-IV	SNRI	10(8/2)	31.8±14.3	serum	17.9±9.1	34.6±7.1	23.2±4.6	8.2±3.9
Shimizu	2003	Japan	DSM-IV	SSRI,SNRI	4(2/2)	46.0±12.2	serum	13.32±11.97	28.79±6.23	33.5±10.5	2.8±1.0

BDNF concentration: Serum, ng/ml; Plasma, pg/ml. SSRI, Selective Serotonin Reuptake Inhibitor. SNRI, selective noradrenalin reuptake inhibitors. TCA, tricyclic antipsychotics.

**Table 2:** BDNF levels of different point times and antidepressant treatment.

Study	Drug group	Dose	Sample	Outcome	BL	1 week	2 week	4 week	6 week	8 week	12 week
Mikoteit	Duloxetine (SNRI)	65.8±16.1mg/day	Serum	BDNF	5.01±3.64	8.66±5.93	11.03±8.0		7.3±5.6		
				HDRS	22.2±4.9	16.8±7.5	13.8±7.5		9.0±6.6		
Ghosh	Fluoxetine (SSRI)	20mg/day	Plasma	BDNF	775.32±30.38				N/A		850.3±24.92
				HDRS	19±4.09				12.2±4.58		9.24±3.98
	Venlafaxine (SNRI)	50mg/day	Plasma	BDNF	760.5±28.53				N/A		845.8±32.82
				HDRS	18±3.75				13.5±3.86		10±3.75
Yoshimura	Paroxetin (SSRI)	30.5±12.4 mg/day	Serum	BDNF	8.6±7.2			9.2±6.1		11.2±8.4	
	Sertraline (SSRI)	76.7±24.0mg/day		HDRS	21.0±4.3			13.8±4.9		10.0±5.6	
	Fluvoxamin (SSRI)	100±26.3 mg/day									
Martocchia	Escitalopram (SSRI)	10mg/day	Serum	BDNF	11.5±0.6					16.0±2.7	
				GDS	9.6±3.4					6.4±3.6	
Brunoni	Sertraline (SSRI)	50mg/day	plasma	BDNF	1331±786					1620±672	
				HDRS	22.0±4.0					14.0±8.0	
Munno	Paroxetin (SSRI)	20-50mg/day	Serum	BDNF	14.87±2.9					14.69±1.6	
				HDRS	19.20±1.83					12.44±2.40	
			Plasma	BDNF	231.45±90.08					312.95±109.42	
				HDRS	19.20±1.83					12.44±2.40	
Ladea	Escitalopram(SSRI)	10mg/day	Serum	BDNF	35.6±12.7			34.7±11.8			35.7±13.2
				MADRS	29.6±2.3						
Deuschle	Venlafaxine (SNRI)	203±47mg/day	Serum	BDNF	7.82±3.75			7.18±5.64			
				HDRS	23.0±4.5			7.4±5.3			
	Mirtazapine	46±9mg/day		BDNF	7.64±6.23			8.50±5.37			
				HDRS	22.7±4.2			8.9±6.0			
Dreimüller	SSNRI, SNRI, TCA, Mirtazapine, Tranylcypromine	N/A	Plasma	BDNF	298±196	334±157					
				HDRS	19	12					
Wolkowitz	Escitalopram (SSRI)	10-20mg/day	Serum	BDNF	15.28 ± 3.55					17.46 ± 3.96	
				HDRS	31.2 ± 7.9					14.5 ± 11.2	
	Sertraline (SSRI)	50-200mg/day		BDNF	14.43 ± 7.12					20.77 ± 10.15	
				HDRS	19.2 ± 4.0					9.7 ± 4.9	
Rojas	Venlafaxine (SNRI)	75mg/day	Serum	BDNF	12.2±9.95				8.85±2.44		
				HDRS	22.8±3.65				11.43±1.48		
Matrisciano	Sertraline (SSRI)	96.4±50.8 mg/day	Serum	BDNF	29.4±12.6				50.6±14.2		
				HDRS	19±5.3				N/A		
	Escitalopram (SSRI)	16.4±3.8 mg/day		BDNF	44.4±16.4				38.6±14.4		
				HDRS	14.3±5.9				N/A		
	Venlafaxine (SNRI)	150±61 mg/day		BDNF	32.3±14.0				29.1±16.3		
				HDRS	19.4±4.5				N/A		
Piccinni	Citalopram, Sertraline, Paroxetine, Amitriptyline, Imipramine, Trimipramine, Desipramine	N/A	Serum	BDNF	19.3±8.8	22.09±8.37	24.46±9.92				
				HDRS	22.8±5.3						
			Plasma	BDNF	290.0±190.0	444.8±20.95	456.3±19.85				
				HDRS	22.8±5.3						
Huang	Mirtazapine. Fluoxetine, Paroxetine, Venlafaxine	N/A	Serum	BDNF	10.7±7.3			12.0±8.9			
				HDRS	35.1±4.9			12.1±10.8			
Hellweg	Amitriptyline (TCA)	150mg/day	Serum	BDNF	13.001±3.744			15.18±5.98			
				HDRS	23.8±5.1			11.0±8.1			
	Paroxetine (SSRI)	40mg/day		BDNF	13.250±5.112			12.02±4.85			
				HDRS	22.6±3.9			11.7±8.4			
Yoshimura	Paroxetine (SSRI)	31±13mg/day	Serum	BDNF	9.4±7.9			10.4±8.2		17.9±7.6	
				HDRS	24±7						
	Milnacipran (SNRI)	83±31mg/day		BDNF	9.8±9.7			10.3±7.5		15.8±8.1	
				HDRS	23±6						
Aydemir	Escitalopram (SSRI)	10 mg/day	Serum	BDNF	27.68±13.74				38.57±15.30		
				HDRS	39.75±7.40				9.31±6.05		
Gonul	Fluoxetine, Venlafaxine	N/A	Serum	BDNF	20.8±6.7					33.3±9.89	
				HDRS	27.28±3.53					8.85±3.15	
Aydemir	Venlafaxine (SNRI)	75 mg/day	Serum	BDNF	17.9±9.1						34.6±7.1
				HDRS	23.2±4.6						8.2±3.9
Shimizu	Amoxapine, Milnacipran, Paroxetine	N/A	Serum	BDNF	13.32±11.97					28.79±6.23	
				HDRS	33.5±10.5					2.8±1.0	

BDNF concentration: Serum, ng/ml; Plasma, pg/ml. SSRI, Selective Serotonin Reuptake Inhibitor. SNRI, selective noradrenalin reuptake inhibitors. TCA, tricyclic antipsychotics.

### Studies of antidepressants types

**Fig 2:**
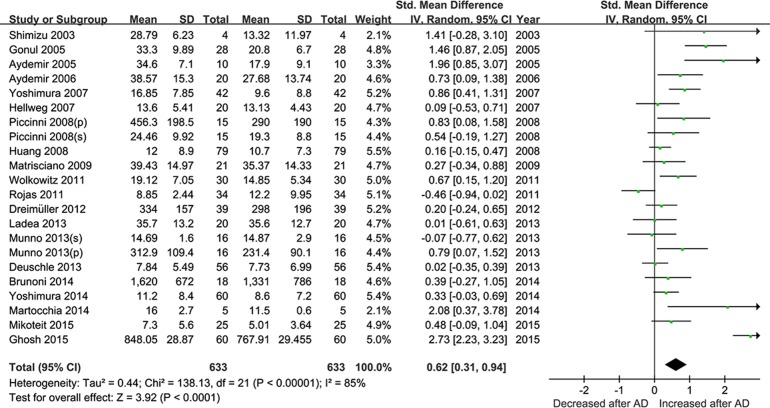
Meta-Analyses for the levels of BDNF pre- and post- antidepressant drugs treatment (less than 12 week).

**Fig 3:**
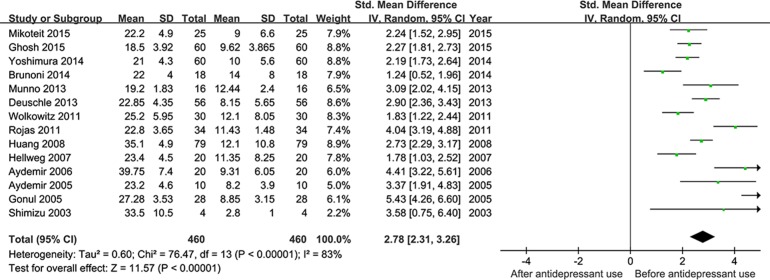
Meta-Analyses for the improvement of depressive symptoms after ADs (less than 12 week).

The twenty studies were pooled together to assess the effect of antidepressant drugs on the BDNF levels before and after treatment. The random effects model was applied, and a pooled analysis of 20 studies showed a significant effect of antidepressants therapy on elevation of BDNF levels (SMD = 0.62, 95% CI = 0.31–0.94, Z = 3.92, *P*<0.0001; **Fig 2**), as well as depressive symptoms amelioration with a significant decreased HDRS score (SMD = 2.78, 95% CI = 2.31–3.26, Z = 11.57, *P* < 0.00001; **Fig 3**). I^2^ were respectively 85%and 83% suggesting strong heterogeneity.

**Fig 4:**
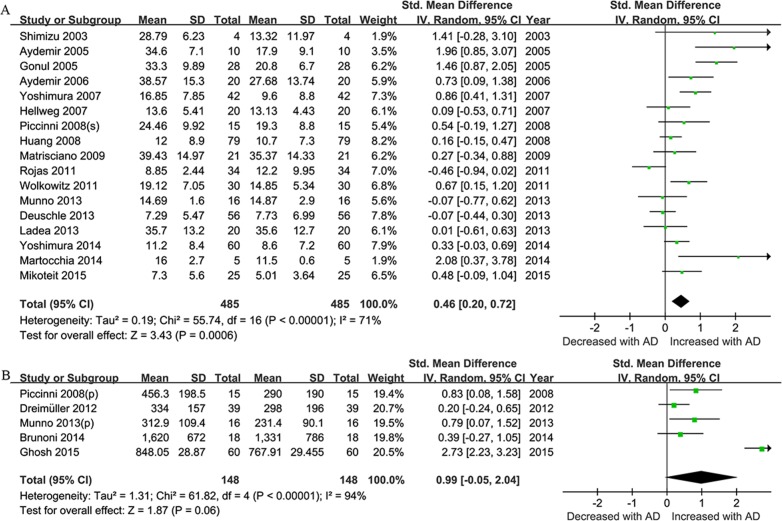
Antidepressants- Stratified Subgroup Meta-Analyses. Forest plots showing the summary effect sizes for levels of BDNF pre- and post- different kind of antidepressant drugs treatment (less than 12 week). (A) using SSRIs;(B) using SNRIs.

**Fig 5:**
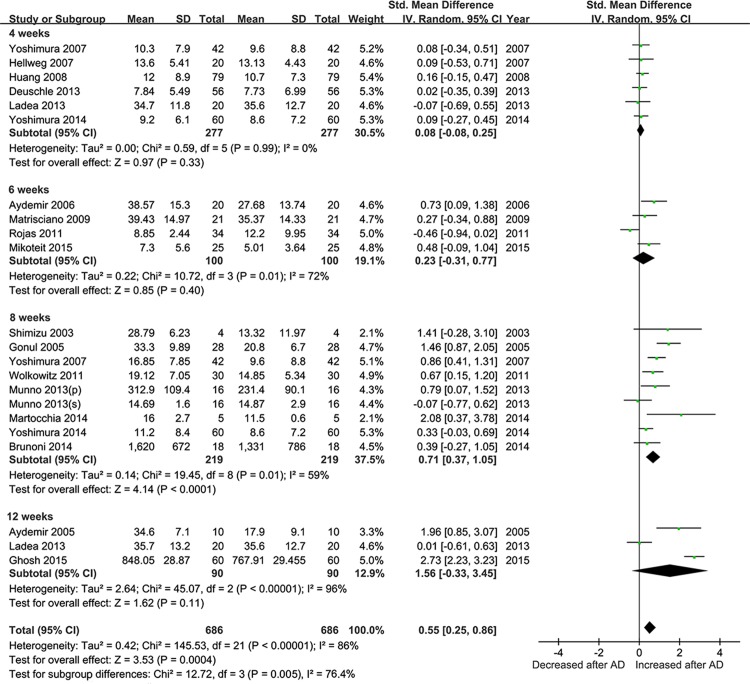
Meta-Analyses for the levels of BDNF pre- and post- antidepressant with differenti drugs treatment (less than 12 week). Forest plots showing summary effect sizes of differential antidepressants (venlafaxine, paroxetine, sertraline and escitalopram) on BDNF concentration changes

We next conducted an analysis from the studies based on the antidepressant drugs use. After stratification with SSRIs and SNRIs, the effect size as the changes in BDNF concentrations pre and post-treatment was 0.68 for SSRIs (95% CI = 0.27–1.10, Z = 3.22, *P* = 0.001; **Fig 4A**), and 0.92 for SNRIs (95% CI = 0.07–1.77, Z = 2.12, *P* = 0.03; **Fig 4B)**. Then efficacy of differential drugs on the BDNF levels after treatment was analyzed. ADs that were only reported in single studies (fluoxetine, duloxetine, mirtazapine, amitriptyline, and milnacipran) or used in combination were excluded. Finally, four types of antidepressants (venlafaxine, paroxetine, sertraline and escltalopram) were analyzed for ease of analysis. According to the SMD and 95%CIs, sertraline showed a statistically significant effect on the BDNF levels pre- and post ADs treatment (SMD = 0.53,95% CI = 0.13–0.93, Z = 2.62, *P*  =  0.009), while no evidence for paroxetine, sertraline and escltalopram in BDNF changes was observed (**Fig 5)**

### Heterogeneity and moderation analyses

**Fig 6:**
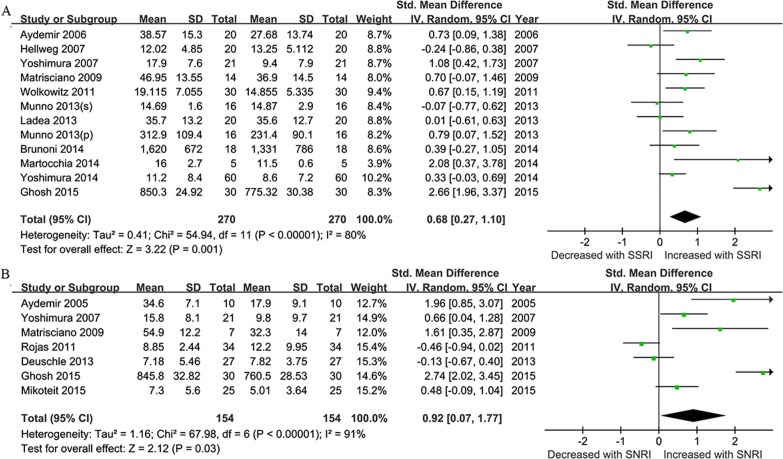
Sample- Stratified Subgroup Meta-Analyses. Forest plots showing the summary effect sizes for levels of BDNF pre- and post- antidepressant drugs treatment (less than 12 week) in serum (A) or plasma(B).

**Fig 7:**
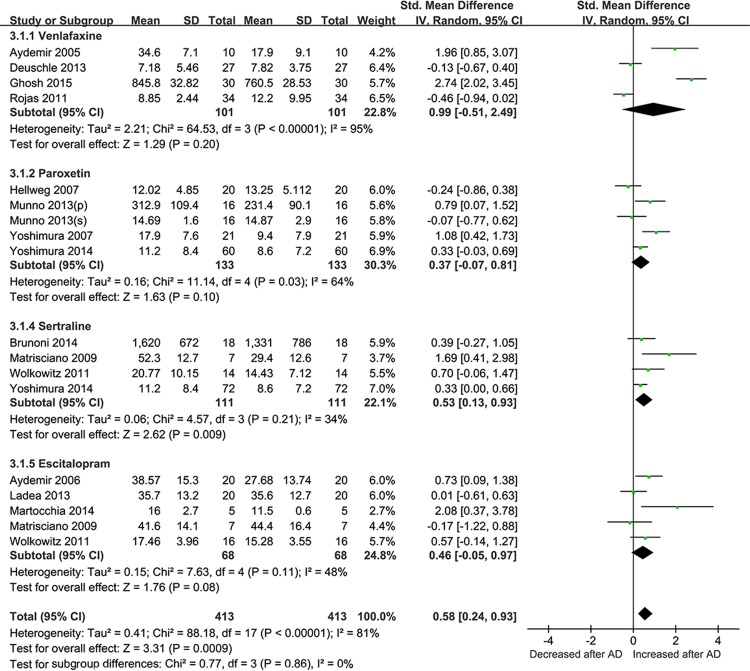
Meta-Analyses for BDNF concentrations at different treatment time points during antidepressants treatment (less than 12 week). Forest plots showing the summary effect sizes for BDNF concentration changes at 4 weeks, 6 weeks, 8 weeks, and 12 weeks.

We performed the subgroup analysis based on the clinical samples used. Of all the included studies, seventeen described the alteration of BDNF levels in serum and five in plasma. The sample-stratified analysis indicated a significant higher level of BDNF concentration post-treatment with an effect-size estimate in serum (SMD = 0.46, 95% CI = 0.20–0.72, Z = 3.43, *P* = 0.0006), which was not observed in plasma (**Fig 6**). In the subgroup meta-analysis based on length of treatment, a statistically significant overall effect size was observed after 8 weeks of antidepressants treatment (SMD = 0.71, 95% CI = 0.37–1.05, Z = 4.14, *P* < 0.0001) for the elevation of BDNF levels **(Fig 7)**.

### Sensitivity and publication bias

Sensitivity analyses were conducted using the leave-one-out method by removing studies one by one to assess the degree to which each individual study influenced the results of the overall analysis. The results of the sensitivity analysis confirmed that no single study influenced the pooled SMDs. No strong statistical evidence for publication bias was observed in Egger׳s test (all *P*>0.05).

## Discussion

In the present study, we summarized the existing literature and conducted a systematic analysis to evaluate the association between BDNF levels and antidepressant drug treatment. We included 20 studies that described comparisons of average BDNF levels pre- and post-antidepressant treatment with SSRIs, SNRIs, TCAs et al. First, we performed systematic meta-analyses and found that subjects with MDD had significantly higher BDNF concentrations after a short-term period (less than 12 weeks) of antidepressants treatment, accompanied by reduced HDRS scores. Consistent with previous works by Molendijk et al, lower serum BDNF in MDD could be normalized during antidepressant treatment [10]. Although we did not discuss whether BDNF concentration was correlated with depressive symptom alleviation, a significant negative relevance between depressive symptom alleviation and BDNF concentrations has been reported [13].

Molendijk et al. previously observed that the use of SSRIs was related to the increase in serum BDNF levels, which suggested that increases in serum levels of BDNF during antidepressant treatment appear to be confined to some antidepressants [10]. To uncover potential differences between various classes of antidepressants, we compared BDNF levels in MDD after SSRIs and SNRIs treatment. In our present study, we found that not only SSRIs but also SNRIs could increase BDNF levels over the course of antidepressant treatment. Making comparisons of four ADs by performing meta-analyses, only sertraline (but not venlafaxine, paroxetine or escitalopram) increased the BDNF levels. Fluoxetine was not analyzed because there was only one study that reported detailed data involving fluoxetine and BDNF.

Length of antidepressants treatment from 4 to 12 weeks was analyzed for contributions to the origin of the heterogeneity in our subgroup meta-analysis, and antidepressants had significant effect on the increase in BDNF concentration after 8 weeks of treatment. Notably, the temporal correlation between serum BDNF levels and the antidepressant effect is not direct: ketamine and ECT treatment increase serum BDNF levels only gradually while their antidepressant effect appears to be quick. While response to ADs treatment in depression was mostly chronic, with subjects in most studies still presenting with depressive symptoms at follow-up, the effect of antidepressant drugs on the BDNF levels may take a long time [40].

After stratifying the subgroup analyses based on clinical blood sampling, serum BDNF levels (as opposed to plasma levels) increased significantly after ADs treatment, while the plasma BDNF levels did not show significant differences; however, a high level of heterogeneity still existed. The significances of the lower BDNF levels in the peripheral blood from subjects with depression were recently reported worldwide in many clinical studies. However, the underlying mechanisms remained unclear. BDNF in the plasma or serum could be released from blood platelets [41, 42]; on the other hand, BDNF could be derived from the brain across the blood-brain-barrier (BBB)[43,44]. Increases in serum BDNF levels with antidepressant treatment have been reported in multiple human and preclinical studies. Chronic administration of ADs could increase the expression of BDNF mRNA and protein in the prefrontal cortex, hippocampus, and other emotion-processing related regions [45–47]. In the overall meta-analysis, we found that the administration of ADs could alleviate the depressive symptoms, and increase the BDNF levels after a time period ranging from 2 to 12 weeks, indicating that changes in serum BDNF levels were significantly correlated with antidepressant treatment. Our subgroup-analysis results suggest that serum BDNF levels increased over the course of antidepressant treatment which was consistent with previous work, while plasma BDNF levels were not observed to be correlated with antidepressant treatment. Our results showed that the detection of BDNF in serum appears to be more reliable.

Mature brain-derived neurotrophic factor (BDNF) is initially synthesized and converted from its precursor protein proBDNF by tPA and extracellular proteases such as matrix metalloproteinase-9 (MMP-9)[48–50]. Both mature BDNF and proBDNF were proved to have important biological function in the pathology of psychiatric disorders such as MDD [51–53]. Mature BDNF and proBDNF in human blood could be distinguished with specific antibody by Western Blotting method. In fact, peripheral BDNF levels were mostly measured using an unspecific antibody BDNF ELISA kits for a larger sample size more conveniently and accurately. However, it was difficult to distinguish between proBDNF and mature BDNF in the ELISA kits due to the limited specificity of the BDNF antibody. A work by Yoshida et al. found that serum levels of mature BDNF, but not proBDNF were significantly lower in patients with MDD [54]. Serum mature BDNF levels in the MDD patients were significantly lower than those in the healthy controls, while no difference was found in serum proBDNF [55]. Therefore, it is likely that mature BDNF contributes to the decreased levels of BDNF in patients with MDD [17].

There exist some possible determinants that could influence serum BDNF. Serum BDNF concentrations systematically vary over the year, and decrease significantly over the autumn-winter period (Jan to May) while increasing in the spring-summer period [56]. Furthermore, other factors such as sampling, the number of sunlight hours, socio-demographics, lifestyle indicators, and disease can influence BDNF levels in humans [56–57]. In our meta-analyses, because of the limited information obtained from each included study, only the sample using and length of treatment duration were analyzed and shown to be potential factors that contribute to the pooled effects and the heterogeneity between studies. Nonetheless, serum levels of BDNF in humans may be influenced by seasonal variation, sex, sunshine hours, blood withdrawal, storage, smoking status, and food and alcohol intake [10, 56, 57].

There are several other limitations with respect to our findings. Firstly, we considered a treatment period within 12 weeks for BDNF analysis. Some ADs may take longer to affect BDNF levels once the depressive symptoms have improved. Secondly, BDNF is a hot topic in depression research worldwide. We only included the English studies in our meta-analysis, which may be a source of publication bias although no such publication bias was found in our meta-analysis. Thirdly, some ADs such as fluoxetine, duloxetine, mirtazapine, amitriptyline, and milnacipran were only reported in single study, so it was difficult to estimate their true effects on the increase of BDNF levels in our analyses. It is important to point out here that these drugs were reported to increase BDNF levels in their respective studies. In some clinical studies, drugs were used in combination which may explain the observed heterogeneity. In addition, drug dose of antidepressant should be analyzed as a modifier for changes of BDNF levels. Due to the limited information and a wide variation in dose, it is difficult to define and divide the dose, so the analysis was not able to be carried out.

In summary, our meta-analyses of 20 studies related to depression provided evidence that peripheral BDNF levels are increased during the course of antidepressant drug treatment (SSRIs and SNRIs). Our study also provided further evidence of the possible mechanisms and effects of different ADs on BDNF levels. Peripheral BDNF levels are better documented in serum than in plasma which would be useful in future research studies of BDNF and antidepressant treatment. Sertraline was shown to increase BDNF levels after a short-term period of treatment, and may be a good candidate for the exploration of ADs pharmacological mechanisms related to peripheral BDNF.

## Supporting information

S1 Text PRISMA Checklist. (DOC)

S2 Text Search strategy used to identify the included studies. (DOC)
