# Supplementary material for: Meta-analyses of comparative efficacy of antidepressant medications on peripheral BDNF concentration in patients with depression
Source: PLoS One. 2017 Feb 27;12(2):e0172270. doi: 10.1371/journal.pone.0172270 (PMC5328267; doi:10.1371/journal.pone.0172270)
Supplement: S2 Text — (DOC) [file pone.0172270.s002.doc]

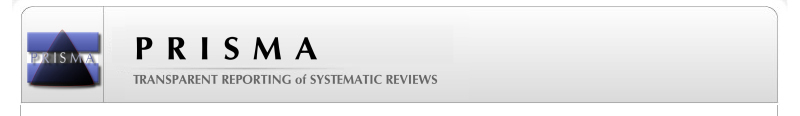
**PRISMA 2009 Flow Diagram**

**Screening**

**Included**

**Eligibility**

**Identification**

Records identified through searching PsycINFO, PubMed, EMBASE, Cochrane, Web of Science with the key-words: BDNF OR brain derived neurotrophic factor AND depression OR MDD OR major depressive disorders OR antidepressant (n=209)

Duplication were detected and excluded (n=104)

49 records were excluded after titles and abstracts review

56 full-text records were reviewed for eligibility

20 papers were included for network meta-analysis

36 full-text records were excluded:

Duplicated cohorts (n=2)

Reviews or meta-analysis (n=5)

BDNF measuring not pre- and post- antidepressants treatment (n=7)

Comparison of BDNF val66met SNP (n=6)

Comparison of ECT on BDNF (n=8)

Comparison of sleep deprivation therapy on BDNF (n=1)

Comparison of exercise on BDNF (n=1)

Data not obtained (n=2)

Measuring BDNF not in plasma or serum (n=2)

Comparison of augmentation effect (n=2)
